# The effect of resveratrol on beta amyloid-induced memory impairment involves inhibition of phosphodiesterase-4 related signaling

**DOI:** 10.18632/oncotarget.8041

**Published:** 2016-03-13

**Authors:** Gang Wang, Ling Chen, Xiaoyu Pan, Jiechun Chen, Liqun Wang, Weijie Wang, Ruochuan Cheng, Fan Wu, Xiaoqing Feng, Yingcong Yu, Han-Ting Zhang, James M. O'Donnell, Ying Xu

**Affiliations:** ^1^ Department of Clinical Pharmacy, Hangzhou First People's Hospital, Hangzhou, Zhejiang Province, China; ^2^ Department of Thyroid Surgery, Kunming Medical University Affiliated First People's Hospital, Kunming, Yunnan Province, China; ^3^ Department of Neurology, Lianyungang Second People's Hospital, Lianyungang, Jiangsu Province, China; ^4^ School of Pharmaceutical Engineering and Life Sciences, Changzhou University, Changzhou, Jiangsu Province, China; ^5^ Department of Pharmaceutical Sciences, School of Pharmacy and Pharmaceutical Sciences, State University of New York at Buffalo, Buffalo, New York, United States of America; ^6^ Brain Institute, School of Pharmacy, Wenzhou Medical University, Wenzhou, Zhejiang Province, China; ^7^ Department of Behavioral Medicine and Psychiatry, West Virginia University, Morgantown, West Virginia, United States of America

**Keywords:** resveratrol, beta amyloid peptide, learning and memory, PDE4, apoptosis, Gerotarget

## Abstract

Resveratrol, a natural polyphenol found in red wine, has wide spectrum of pharmacological properties including antioxidative and antiaging activities. Beta amyloid peptides (Aβ) are known to involve cognitive impairment, neuroinflammatory and apoptotic processes in Alzheimer's disease (AD). Activation of cAMP and/or cGMP activities can improve memory performance and decrease the neuroinflammation and apoptosis. However, it remains unknown whether the memory enhancing effect of resveratrol on AD associated cognitive disorders is related to the inhibition of phosphodiesterase 4 (PDE4) subtypes and subsequent increases in intracellular cAMP and/or cGMP activities. This study investigated the effect of resveratrol on Aβ1-42-induced cognitive impairment and the participation of PDE4 subtypes related cAMP or cGMP signaling. Mice microinfused with Aβ1-42 into bilateral CA1 subregions displayed learning and memory impairment, as evidenced by reduced memory acquisition and retrieval in the water maze and retention in the passive avoidance tasks; it was also significant that neuroinflammatory and pro-apoptotic factors were increased in Aβ1-42-treated mice. Aβ1-42-treated mice also increased in PDE4A, 4B and 4D expression, and decreased in PKA level. However, PKA inhibitor H89, but not PKG inhibitor KT5823, prevented resveratrol's effects on these parameters. Resveratrol also reversed Aβ1-42-induced decreases in phosphorylated cAMP response-element binding protein (pCREB), brain derived neurotrophic factor (BDNF) and anti-apoptotic factor BCl-2 expression, which were reversed by H89. These findings suggest that resveratrol reversing Aβ-induced learning and memory disorder may involve the regulation of neuronal inflammation and apoptosis *via* PDE4 subtypes related cAMP-CREB-BDNF signaling.

## INTRODUCTION

Alzheimer's Disease (AD) is a neurodegenerative disorder characterized by accumulation of beta amyloid peptides (Aβ) and neurofibrillary tangles (NFTs) in the brain, widespread cortical neuronal loss and the progressive memory impairment [1]. The accumulation of Aβ, particularly Aβ 1-40 (Aβ 40) and Aβ1-42 (Aβ 42), and their deposition in insoluble plaques are the major neuropathological hallmarks of AD. It is suggested that the form of Aβ 42 is more neurotoxic than that of Aβ 40 [2]. Injection of plaques of Aβ 42 isolated from AD brains into different brain regions of rats including hippocampus and cortex results in neurodegenerative response, such as neuroinflammation and cell apoptosis [3]. Although the contradictory results argue the role of Aβ in the onset of AD [4], inhibition of cerebral Aβ 42 seems necessary and sufficient for preventing memory impairment in the early stage of AD. However, the specific mechanisms causing AD and memory deficits remain arguing due to lack of scientifically proven pharmacological strategies. Therefore, it is urgent to identify new targets and develop novel anti-aging agents against AD.

Resveratrol is a natural polyphenol extracted from grapes in the processing of red wine. It has been found to exert numerous pharmacological properties including antioxidative, antiinflammatory and antiaging activities. Recent studies suggested resveratrol could protect hippocampal neurons against Aβ 40 and oxidative stress-induced neurotoxicity and cell death [5, 6], suggesting its potential role in treatment of aging-related learning and memory deficits. Importantly, some studies suggested that resveratrol increases cAMP production in breast cancer cells [7]; cAMP levels were also found to increase in skeletal muscle and white adipose tissue after treatment with low doses of resveratrol [8]. Since intracellular cAMP levels are usually controlled by phosphodiesterase (PDE) activities, the further study found that resveratrol is a PDE inhibitor. Recently, the role of phosphodiesterase 4 (PDE 4) inhibitors in learning and memory performance has provoked intense interest in discovery of small-molecular components from natural polyphenols that could delay or halt Aβ-related cognitive disorders by inhibition of PDE4. It keeps mysterious whether the increased cAMP levels by treatment with resveratrol are related to inhibition of PDE4 and its subtypes in the hippocampus, and whether this inhibition of PDE4 dependent pathway contributes to the subsequent anti-inflammatory and neuroprotective effects, as well as the cognitive enhancement in behavior.

The present study attempted to find the memory enhancing effect of resveratrol on cognitive impairment induced by Aβ 42, and to elucidate the biological pathway by which it inhibits PDE4 subtypes and stimulates cAMP-related neuroprotective effects. We found that resveratrol inhibits cAMP-specific PDE4A, 4B and 4D, and the related signaling in the hippocampus that mediates the anti-neuroinflammatory and anti-apoptotic effects, and finally results in the memory enhancing effects.

## RESULTS

### Resveratrol reversed Aβ-induced memory impairment in the Morris water maze task

**Figure 1:**
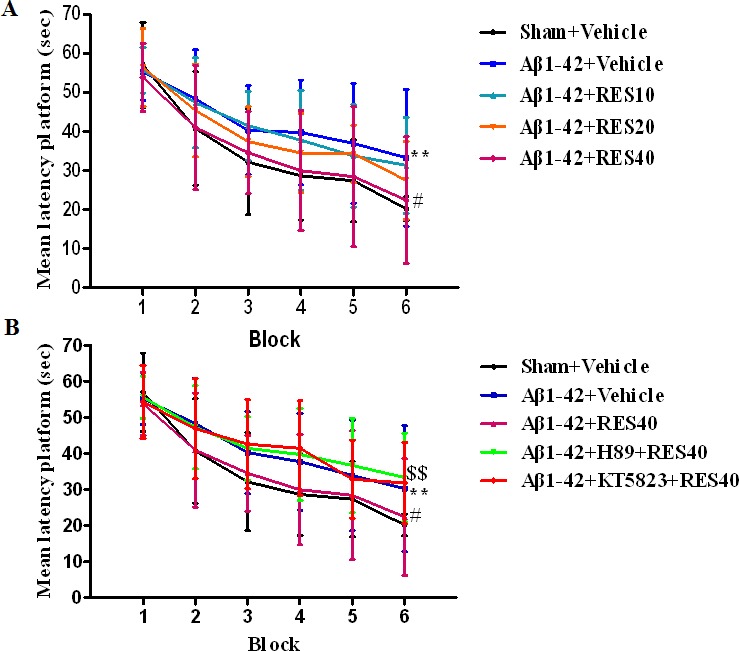
Learning curve in the water maze task of vehicle-treated sham group and Aβ-treated mice administered with vehicle, resveratrol (10, 20 and 40 mg/kg), H89 and KT5823 Seven days after microinfusion with Aβ, mice were administered with resveratrol for 21 days. H89 and KT5823 were pretreated 30 min before resveratrol administration every day. Behaviors were tested 30 min after last treatment (mean±SEM, *n* = 10). **p < 0.01 *vs.* vehicle-treated sham group. ^#^*p* < 0.05 *vs.* vehicle-treated Aβ group. ^$$^*p* < 0.01 *vs.* RES40-treated Aβ group.

To determine whether resveratrol reversed memory impairment caused by Aβ 42, we examined memory performance in the Morris water maze test in mice treated with Aβ 42 in the presence or absence of resveratrol. Aβ42 (0.4 μg/side) was infused into bilateral CA1 subregion of the hippocampus. Although all mice reliably learned to locate the platform throughout 6 blocks of acquisition training, the groups significantly differed in their latency to reach the platform during the 6 training blocks. The results showed that vehicle-treated Aβ group mice took longer to reach the platform in block 6 compared with vehicle-treated sham group mice (*p* < 0.01), these were reversed by resveratrol at 40 mg/kg (RES40) for 21 days, the latencies to reach the platform for RES40-treated Aβ mice were significantly shorter than that of the vehicle-treated Aβ group in the 6^th^ block (*p* < 0.05; Figure 1A). While these effects of resveratrol (40 mg/kg) on acquisition were blocked by pretreatment with PKA inhibitor H89 (*p* < 0.01; Figure 1B). However, the PKG inhibitor KT5823 did not reverse the effect of resveratrol. H89 and KT5823 did not have effects on Aβ-induced impairment of acquisition when they were treated alone (data not shown).

**Figure 2:**
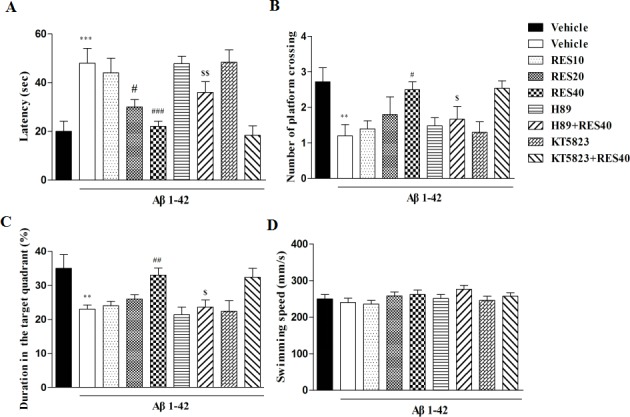
Resveratrol reversed A-induced memory impairment 1 h after the training session Latency to reach the platform **A.** number of platform crossing **B.** duration in the target quadrant **C.** and swimming speed **D.** during the 1 h probe trials of the water maze were shown after treatment with resveratrol for 21 days (mean±SEM, *n* = 10). **p < 0.01, ^***^p < 0.001 *vs.* vehicle-treated sham group. ^#^*p* < 0.05, ^##^*p* < 0.01 and ^###^*p* < 0.001 *vs.* vehicle-treated Aβ group. ^$^*p* < 0.05 and ^$$^*p* < 0.01 *vs.* RES40-treated Aβ group.

One hour after the training, spatial memory was assessed in the probe trial test, during which the platform was removed. Vehicle-treated Aβ group mice showed significantly longer latencies to reach the platform, made fewer crossings over the platform, and altered duration in the target quadrant compared with vehicle-treated sham group (*p* < 0.001; *p* < 0.01 and *p* < 0.01, respectively). RES40-treated Aβ mice took significantly less time to reach the platform, made more crossings over the platform, and more exploration time in the target quadrant than vehicle-treated Aβ group (F(3,36) = 3.36), *p* < 0.001; F(3,36) = 3.993), *p* < 0.05; F(3,36) = 2.863), *p* < 0.01; respectively). These could be reversed by pretreatment with H89, but not KT5823 (Figure 2A-2C).

**Figure 3:**
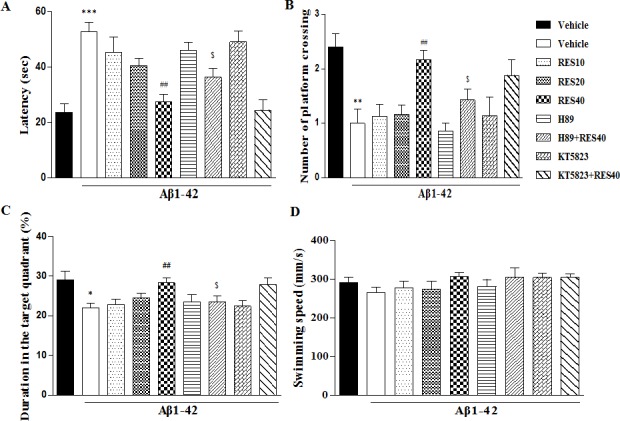
Resveratrol reversed Aβ-induced memory impairment 24 h after the training session Latency to reach the platform **A.** number of platform crossing **B.** duration in the target quadrant **C.** and swimming speed **D.** were shown during the 24 h probe trials of the water maze after treatment with resveratrol for 21 days (mean±SEM, *n* = 10). *p < 0.05, **p < 0.01 and ^***^p < 0.001 *vs.* vehicle-treated sham group. ^###^*p* < 0.001 *vs.* vehicle-treated Aβ group. ^$^*p* < 0.05, *vs.* RES40-treated Aβ group.

Memory retention for the platform location on the probe trial was tested 24 h after the training section. Similar to that of in the 1 h probe trail test, longer latencies, fewer crossings and exploration time in the target quadrant were detected in the vehicle-treated Aβ group (*p* < 0.001, p < 0.01 and *p* < 0.01, respectively). RES40 ameliorated the detrimental effects of Aβ on platform latency, crossings and the time in the target quadrant [F(3,36) = 7.554, *p* < 0.01; F(3.36) = 5.<638, *p* < 0.01; F(3,36) = 4.031, *p* < 0.01]. However, resveratrol's effects were blocked by pretreatment with H89, but not KT5823 (Figure 3A-3C).

The swim speed was not altered among all the groups in the 1 h or 24 h test, which showed that surgery operation and drug treatment did not affect the animals' abilities of vision and motor activity (Figures 2D and 3D).

### Resveratrol reversed Aβ-induced memory impairment in the step-down passive avoidance task

**Figure 4:**
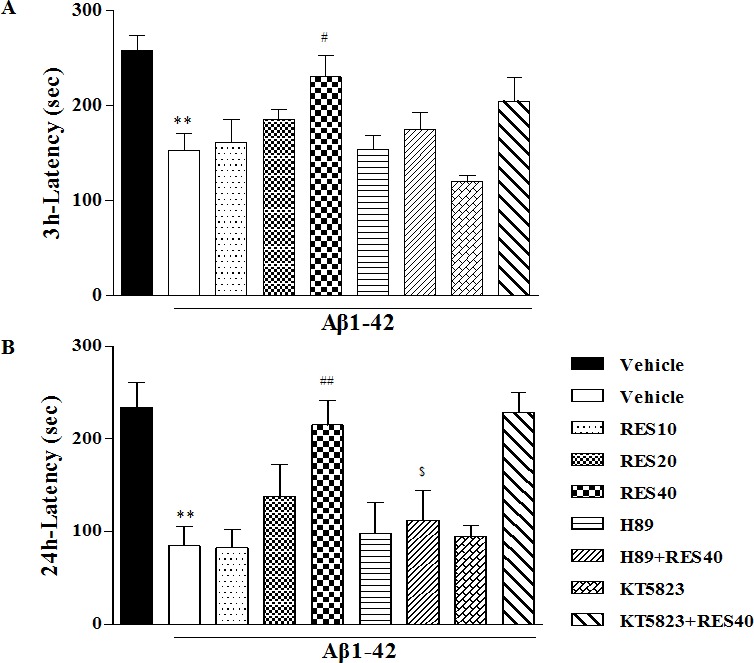
Effects of resveratrol on 3-h **A.** and 24-h **B.** retention in the step-down passive avoidance test in Aβ 42-treated mice. Aβ 42-induced decreases in 3-h and 24-h retention were reversed by chronic treatment with resveratrol for 21 days (mean±SEM, *n* = 10). **p < 0.01*vs.* vehicle-treated sham group. ^#^*p* < 0.05 and ^##^*p* < 0.01 *vs.* vehicle-treated Aβ group. ^$^*p* < 0.05,*vs.* RES40-treated Aβ group.

To confirm the reversal effect of resveratrol on memory deficits induced by Aβ42, the mice were tested for memory performance using the step-down passive avoidance task. As shown in Figure 4A, in the retention test performed 3 h after training, Aβ42 induced significant memory impairment, as evidenced by a lower retention when compared with sham group (*p* < 0.01). RES40 administered for 21 days exhibited significantly higher retention (F(3,36) = 4.840, *p* < 0.05), which was blocked by pretreatment with H89 (*p* > 0.05). Meanwhile, retention latency tested 24 h after initial training indicated that Aβ1-42-treated mice exhibited a decrease in retention relative to sham group (*p* < 0.01). This effect was reversed by resveratrol at 40 mg/kg (*p* < 0.01). However, H89 blocked the effect of RES40 significantly (*p* < 0.05), whereas KT5823 did not affect the effect of RES40 (Figure 4B).

### Resveratrol's reversal of Aβ42-induced changes in IL-1β, IL-6, Bcl-2, and Bax levels in the hippocampus

**Figure 5:**
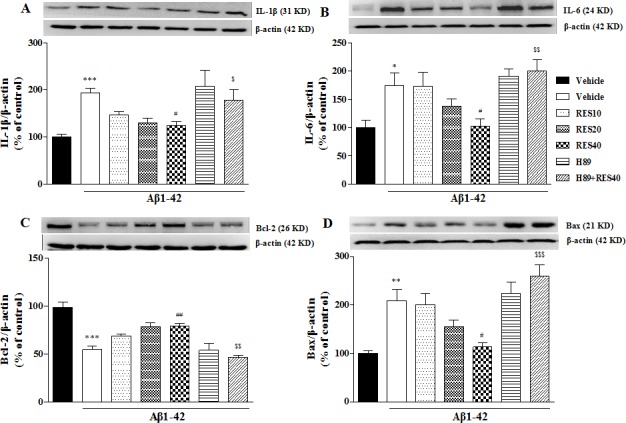
Effects of resveratrol on Aβ 42-induced changes in IL-1β **A.** IL-6 **B.** Bcl-2 **C.** and Bax **D.** in the hippocampus of mice (mean±SEM, *n* = 10). *p < 0.05, **p < 0.01, and ^***^p < 0.001 *vs.* vehicle-treated sham group. ^#^*p* < 0.05 and ^##^*p* < 0.01 *vs.* vehicle-treated Aβ group. ^$^*p* < 0.05, ^$$^*p* < 0.01 and ^$$$^*p* < 0.001 *vs.* RES40-treated Aβ group.

To verify the effects of resveratrol on Aβ42-induced changes in neuroinflammatory and apoptotic responses in the hippocampus, we examined expression of pro-inflammatory cytokines, such as IL-1β and IL-6. The IL-1β and IL-6 levels in the vehicle-treated Aβ group were significantly increased when compared with that of the vehicle-treated sham group (*p* < 0.001 and *p* < 0.05). These increases in IL-1β and IL-6 levels were significantly reversed by chronic treatment with resveratrol [F(3,12) = 3.295, *p* < 0.05; F(3,12) = 3.207, *p* < 0.05]. Resveratrol decreased the IL-1β and IL-6 levels, which were prevented by pretreatment with H89 (*p* < 0.05 and *p* < 0.01) (Figure 5A and 5B). In addition, we examined expression of Bax and Bcl-2, both were cell death associated proteins, to determine whether apoptotic responses were involved in the effects of resveratrol on Aβ42-induced toxicity. As shown in Figure 5C and 5D, expression of Bcl-2 and Bax were changed after Aβ42 treatment, i.e. Aβ42 decreased Bcl-2 expression (*p* < 0.001) and increased Bax levels (*p* < 0.01) in the hippocampus. These effects were reversed by treatment with resveratrol at dose of 40 mg/kg (*p* < 0.01 in Bcl-2; *p* < 0.05 in Bax). H89 blocked the effects of resveratrol on Bcl-2 and Bax levels when compared with RES40-treated Aβ group (*p* < 0.01 in Bcl-2; *p* < 0.001 in Bax).

### Effects of resveratrol on cAMP protein kinase (PKA) and PDE4 subtypes levels in the hippocampus

**Figure 6:**
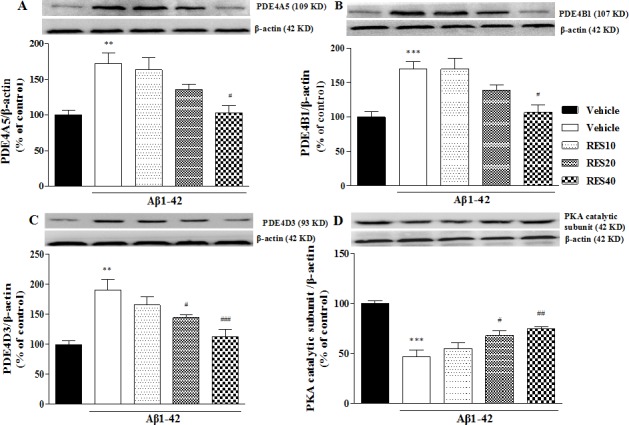
Effects of resveratrol on PDE4A, PDE4B, PDE4D variants and PKA catalytic subunit D expression in the hippocampus of Aβ 42-treated mice (mean±SEM, *n* = 10). **p < 0.01 and ^***^p < 0.001 *vs.* vehicle-treated sham group. ^#^*p* < 0.05, ^##^*p* < 0.01 and ^###^*p* < 0.001 *vs.* vehicle-treated Aβ group.

To determine whether microinfusion of Aβ42 into hippocampus affected PKA and cAMP-related PDE4 expression, we determined the expression of PKA and PDE4 subtypes in the hippocampus. Specific polyclonal PDE4A5, 4B1 and 4D3 antibodies revealed that the major protein bands of molecular masses 109 (for PDE4A5), 107 (for PDE4B1) and 95 kDa (for PDE4D3) in the hippocampus, respectively (Figure 6A, 6B and 6C). The significant increases in PDE4A5, 4B1 and 4D3 expression induced by Aβ42 were found in the hippocampus; while PKA was decreased in the Aβ42-treated mice (*p* < 0.01; *p* < 0.001; *p* < 0.01; *p* < 0.001). Resveratrol at 40 mg/kg reversed the increased expression of PDE4A5 and 4B1 (F(3,12) = 4.779, *p* < 0.05; F(3,12) = 5.386, *p* < 0.05); while resveratrol at 20 and 40 mg/kg reversed the changes of PDE4D3 and PKA levels induced by Aβ42 (F(3,12) = 7.713, *p* < 0.05, *p* < 0.01; F(3,12) = 6.176, *p* < 0.05; *p* < 0.01) (Figure 6C-6D).

### The effects of resveratrol on Aβ1-42-induced decreases in pCREB/CREB and BDNF expression

**Figure 7:**
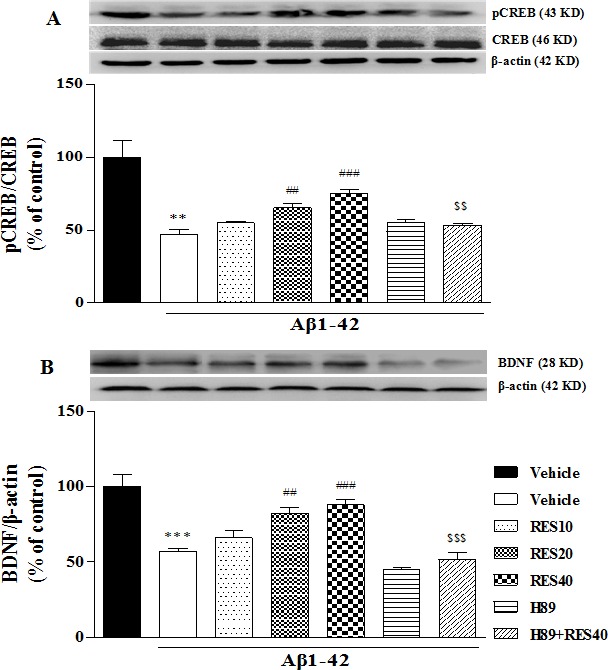
Effect of resveratrol on Aβ 42-induced changes in the ratio of pCREB/CREB **A.** and BDNF **B.** expression in the hippocampus of mice (mean±SEM, *n* = 10). **p < 0.01 and ^***^p < 0.001 *vs.* vehicle-treated sham group. ^##^*p* < 0.01 and ^###^*p* < 0.001 *vs.* vehicle-treated Aβ group. ^$$^*p* < 0.01 and ^$$$^*p* < 0.001 *vs.* RES40-treated Aβ group.

It has been suggested that the cAMP/PKA pathway contributes to the phosphorylation of CREB (pCREB) and is responsible for late phases of the memory consolidation processes. We examined expression of pCREB and CREB to verify whether the cAMP/PKA-CREB pathway was involved in the effects of resveratrol on memory. The amount of CREB phosphorylation between the vehicle-treated Aβ42 group and sham group was found to be significantly different, i.e., Aβ42 decreased pCREB expression (*p* < 0.01). This decrease in the pCREB was reversed by treatment with resveratrol at doses of 20 and 40 mg/kg for 21 d (F(3,12) = 20.65, *p* < 0.01 for 20 mg/kg; *p* < 0.001 for 40 mg/kg). H89 blocked the effect of RES40 on pCREB expression (*p* < 0.01) (Figure 7A).

To determine whether resveratrol's effect on memory performance is related to neuroprotective effect. We investigated BDNF expression in the hippocampus in the presence of resveratrol in Aβ42-treated mice. As shown in Figure 7B, BDNF levels were decreased in the hippocampus of vehicle-treated Aβ42 group when compared with the vehicle-treated sham group (*p* < 0.001), which was reversed by 20 and 40 mg/kg resveratrol treatment (F(3,12) = 13.370, *p* < 0.01 for 20 mg/kg; *p* < 0.001 for 40 mg/kg). However, H89 reversed the effect of resveratrol on BDNF expression (*p* < 0.001).

## DISCUSSION

Natural products provide adequate plant sources of new therapeutic phytochemicals for treating aging related disorders, such as AD. Resveratrol and its derivatives are rich in grapes and have multiple therapeutically benefits in the treatment of diseases associated with AD [9]. The neuroprotective effect of resveratrol has recently been reported in a cell model of amyloid beta peptide-induced neurotoxic injury [9]. Although the in vivo study suggested that a combination of resveratrol and a lipid-core nanocapsule-based delivery system prevents Aβ42-induced neurodegenerative process by modulation of neuroinflammation [10], the specific mechanism by which resveratrol exerts anti-aging effects remains elusive. The present study demonstrated that 20 and 40 mg/kg resveratrol (*via* gavage) ameliorated Aβ42-induced learning and memory impairment in the Morris water maze and step-down passive avoidance tests; resveratrol also prevented memory extinction in the presence of Aβ42 in the passive avoidance test. Pretreatment with PKA inhibitor H89, but not PKG inhibitor KT5823, prevented these memory-enhancing effects. Further molecular biological assays suggested that resveratrol inhibited PDE4A5, 4B1 and 4D3 expression, and subsequently increased cAMP levels. Moreover, high doses of resveratrol decreased Aβ42-induced cytokines (IL-1β and IL-6) and pro-apoptotic protein (Bax) expression, which were blocked by pretreatment with H89. The decreased expression of anti-apoptotic protein (BCl-2), transcription factor (pCREB/CREB) and BDNF levels produced by Aβ42 were significantly reversed by resveratrol. These effects were also blocked by pretreatment with H89. The results suggest that PDE4 subtypes related cAMP signaling might participate resveratrol's protective effects against Aβ42-induced neurotoxicity.

Aβ plays an important role in mediating the pathogenesis of AD. Aβ deposition in the brain regions that involves learning and memory performance, such as frontal cortex and hippocampus, has been considered as an early event in the process of AD [11]. There are two main forms of Aβ, Aβ 40 is the more soluble form, while Aβ42 is the primary component of senile plaques although it is found to have less than 5% in AD cases [1]. Aβ42 has been shown to generate NFTs and tau protein hyperphosphorylation in neuronal cultures, which accelerate cognitive deficits in animals [12-14]. Microinjection of Aβ42 into the hippocampus of mice has been recognized as a reliable and stable animal model of AD, which mimics alterations known for AD patients including memory deficits and downregulation of cAMP signaling [11, 15].

Our present study provided promising demonstration for memory impairment induced by microinfusion of Aβ42 into bilateral CA1 of hippocampus, as evidenced by prolonged escape latency in the acquisition trials and decreased exploration in the target quadrant in the water maze task, and decreased memory retention in the passive avoidance test. Resveratrol at higher dose (40 mg/kg) could reverse this learning and memory impairment induced by Aβ42. Interestingly, this effect was blocked by pretreatment with PKA inhibitor H89, while PKG inhibitor did not affect this resveratrol's memory-enhancing effect. This finding was further supported by the subsequent neuronal biological assays, which suggested that resveratrol could reverse inflammatory and apoptotic cytokines expression in the presence of Aβ42. These effects were also blocked by pretreatment with H89. Studies have revealed the important roles of inflammatory damage and apoptosis in the development or progression of cognitive deficits in neurodegenerative disorders, such as AD [16, 17]. Previous studies suggested that natural-occurring polyphenol curcumin inhibits aging-related pro-inflammatory cytokines expression, such as TNF-α and IL-1β, and increases the index of anti-apoptotic and pro-apoptotic factors, by increases in cAMP levels [2, 18]. Recent evidence demonstrated resveratrol and curcumin exert the synergistic anti-inflammatory effects on aging-related diabetes by inhibiting PDEs [19]. Another group of study supports this finding that suggests PDE4 might be the potential target for treating metabolic diseases associated with aging [20]. The present study extends these previous findings that suggested resveratrol at 40 mg/kg decreased Aβ42-induced IL-1β and IL-6 increasing; while these effects were reversed by pre-treatment with H89. Previous studies suggested that pro-inflammatory cytokines exacerbate apoptotic progress [21, 22]. Inhibition of Il-1β and IL-6 not only prevents inflammatory response but also delays neuronal cell apoptosis [22]. It is interesting to know whether resveratrol produces the anti-apoptotic effect in Aβ42-treated mice. Several proteins are related to the progress of apoptosis, such as Bcl-2, Bax, caspase family members, p53 and p21 [23, 24]. Among them, the Bcl-2 family members are the most intriguing proteins in the regulation of apoptotic process. They can be divided into anti-apoptotic members, such as Bcl-2, Bcl-xl, and Bcl-w, and pro-apoptotic members, such as Bax and Bak [11, 25]. It has been suggested that microinjection of Aβ into brain decreases in Bcl-2 and increases in Bax expression that result in neuronal cell death in mice [26]. Our study suggested that resveratrol, at the same doses that prevented Aβ42-induced learning and memory impairment and reversed pro-inflammatory cytokines, also reversed apoptotic response, as evidenced by increase in Bcl-2 level and decrease in Bax expression in the Aβ42-treated mice. However, these anti-neuroinflammatory and anti-apoptotic effects of resveratrol were blocked by pretreatment with H89, further supporting the participation of cAMP-dependent protein kinases in regulation of Aβ-induced inflammation and apoptosis.

The previous studies suggested that dietary intake of resveratrol derivatives have a positive impact on Aβ42-related brain aging [27, 28], and ameliorate aging-related metabolic phenotypes, such as obesity and diabetes, by inhibiting cAMP-degrading phosphodiesterases, which results in increases in cAMP levels [8, 19]. Unfortunately, there were few reports about the relationship between PDE4 subtypes and resveratrol's effects on aging-related disorders, particularly learning and memory deficits. Indeed, the intracellular cAMP levels are determined by either the activities of ACs that synthesize cAMP from ATP, or PDEs that regulate the hydrolysis of cAMP and/or cGMP to AMP and/or GMP, respectively. The present study suggested that cAMP levels were significantly increased with a dose of 40 mg/kg resveratrol administered in Aβ42-treated mice; further study showed that resveratrol reversed Aβ42-induced increase in PDE4A5, 4B1 and 4D3 expression. It is known that there are four different genes encoded PDE4 (PDE4A- 4D), each of which generates multiple splice variants [29]. However, only three of them, PDE4A, 4B and 4D, are involved in learning and memory performance, PDE4C is only found to express in the peripheral system [30]. More specifically, PDE4A and PDE4D may be the major PDE4 subtypes in mediating memory. Comparably, PDE4B may not be important in hippocampal-dependent memory. This appears to be supported by the negative results in the water-maze memory test using mice deficient in PDE4B [31, 32]. Aβ deposition in frontal cortex and hippocampus involves learning and memory performance has been considered as an early event in the process of AD [11]. The present results revealed that the expression of PDE4A5, 4B1 and 4D3 were increased in Aβ 42-treated mice in the hippocampus, which were in agreement with our preliminary data and the previous studies [33, 34]. Indeed, these PDE4 splice variants are closely related to emotion and the learning and memory performance evidenced by previous studies [33, 35]. The present study also suggested that the increased expression of PDE4A5, 4B1 and 4D3 was reversed by pretreatment with resveratrol for 21 days. Considering that PKA inhibitor H89 blocked resveratrol's effects on memory performance in Aβ 42-treated mice, the present findings give raise the possibility that resveratrol activates cAMP-related signaling by inhibiting PDE4 subtypes, particularly PDE4A, 4B and 4D.

The increase of hippocampal Bcl-2 after treatment with resveratrol in the present study may be explained by the triggering of a neuroprotective pathway that aims to promote neuroregeneration. Indeed, neurons in the hippocampus of aged animals respond to Aβ-induced toxicity by showing atrophy and a down-regulation of BDNF and pCREB expression that are associated with learning and memory impairment [36]. Results from our study suggested that Aβ-induced decreased expression in BDNF and pCREB in the hippocampus of mice. The potential mechanisms for resveratrol's neuroprotective effects might be related to cAMP-signaling, as evidenced by the fact that PKA inhibitor H89 prevented resveratrol's effects on BDNF and pCREB expression. Recent studies have turned the attention to the neuroprotective-neurorescue therapies for AD treatment, which indicate the anti-apoptotic and neuroprotective effects of resveratrol are crucial either exogenously supplied or as endogenously induced elements that eliminate neuronal degeneration.

Taking together, the present study investigated the effects of resveratrol on Aβ42 induced learning and memory impairment and the underlying mechanism. The effects of resveratrol on cognitive behaviors and the subsequent inflammatory cytokines, apoptosis-related proteins, as well as transcription factor and neurotrophins expression support the fact that resveratrol produces anti-aging effects *via* PDE4 subtypes mediated cAMP-dependent pathway.

## MATERIALS AND METHODS

### Animals

Male ICR mice weighing between 22 and 25 g (2-3-month-old) were obtained from Animal Center of Shanghai Branch, Chinese Academy of Sciences. Upon arrival, mice were housed five per cage under standard colony conditions, with controlled ambient temperature (22±1 °C), humidity (50±10 %) and a natural light/dark cycle (12:12h, lights on 7:00 AM). All experiments were carried out according to the National Institutes of Health Guide for Care and Use of Laboratory Animals. Experimental procedures were approved by the Wenzhou Medical University Committee on Animal Care and Use.

### Surgery

The surgical procedure was performed aseptically under chloral hydrate anesthesia. Mice were placed in a stereotaxic apparatus. Two holes are drilled on the skull based on the coordinates for hippocampal CA1 (AP −1.7 mm from bregma, ML ±0.8 mm from midline, DV −2.0 mm from dura) before a guide cannula (30-gauge) was inserted in each hole and fixed in place. After surgery, the mice were allowed to recover for 3 days. Mice were given bilateral microinjections of 2 μl Aβ42 (0.4 μg in 1 μl/side, rPeptide, USA), corresponding volumes was infused bilaterally at the rate of 0.25 μl/min using a syringe pump. The doses of Aβ42 based on the previous research [16]. The mice in sham group were given bilateral microinjections of 2 μl artificial cerebrospinal fluid.

### Drugs and treatments

Aβ42 (rPeptide, USA) was dissolved in 0.9% sterile saline, at a final concentration of 0.4 mg/ml. The Aβ42 solution was incubated at 37 °C for 4 days to obtain aggregated Aβbefore use [37, 38]. Resveratrol (Sigma-Aldrich) was prepared daily by dissolving in 0.9% sterile saline. The mice received different doses of resveratrol (10, 20 and 40 mg/kg, p.o.) or vehicle for 3 weeks after bilateral microinjections of Aβ42.

KT5823 and N-[2-(p-bromocinnamylamino)ethyl]-5-isoquinolinesulfonamide (H-89) (Sigma-Aldrich) were dissolved in artificial cerebrospinal fluid. KT5823 (20 μM) and H89 (5 μM) were administered 30 min before treatment with resveratrol. Mice were given microinjection of 2 μl drugs (1 μl/side) into the CA1 of the hippocampus.

### Behavioral test procedures

To examine the effect of resveratrol on Aβ42-induced memory deficits, 10 mice each group were tested for memory using Morris water maze and passive avoidance tasks: (a) saline+vehicle; (b) Aβ42+vehicle; (c) Aβ42+resveratrol (10, 20, 40 mg/kg); (d) Aβ42+H89; (e) Aβ42+H89+resveratrol (40 mg/kg); (f) Aβ42+KT5823; (g) Aβ42+KT5823+resveratrol (40 mg/kg). Fourteen days after microinfusion of Aβ42, mice were treated with different doses of resveratrol or vehicle, once per day for 3 weeks before Morris water maze and passive avoidance tests were performed. The behavioral tests were subjected 30 min after the last drug treatment.

### Morris water maze test

This was performed as described previously [32, 39] with slight modifications. The apparatus consisted of a circular pool (95 cm diameter, 25 cm height) filled with water, which was opaque by mixing with milk powder, and a platform, which was either visible or immersed 1cm under the surface of the water in one of the four identical quadrants. The mice were taken for the prior habituation to the pool one day before the test. The acquisition trials (training to escape to the hidden platform) were carried out for six blocks consisting of three (60 s) trials separated by 20 min inter-block intervals during which the platform remained in the same location relative to the distal cues in the room. On each trial, mice were placed in the pool each day from different starting points (E, S, W and N). If mice failed to find the platform within 60 s, mice were guided to the platform manually. One hour after the last acquisition trial, the probe trial test was conducted with the platform removed. Swimming behaviors, including escape latency (time spent in locating the platform), entries in target quadrant, duration in target quadrant, swim distance, and swimming speed were monitored using a computer controlled video-tracking system (CG-400 Image Acquisition System, Institute of Materia Medica, Chinese Academy of Medical Sciences, Shanghai, China). Another probe trial was run 24 h after training to assess consolidation and retrieval of memory [40].

### Step-down passive avoidance test

It was carried out in mice using a chamber containing a wooden platform on one side of the grid floor [41], electric shocks were delivered to the grid using an isolated pulse stimulator. During the training, mice were individually placed on the platform and subjected to a foot shock (0.4-0.8 mA, 40 V, 0.5 s, 50 Hz, 20 sec intertrial interval) when they completely descended to the grid floor. This procedure was repeated immediately and again 1 h after the initial training. Mice that stayed on the platform for over 60 s were considered to have learned the task and were removed to their home cages, without being given further shocks. Retention tests were carried out 3 h and then 24 h after the last training session. For all retention tests, each mouse was placed on the platform and the step-down latency was recorded, with an upper cut-off time of 300 s.

### Western blot analysis

Mice were decapitated after passive avoidance test, the hippocampus were dissected and stored at −80 °C until analysis. The hippocampal tissues were homogenized in RIPA lysis buffer containing protease and phosphatase inhibitors and centrifuged at 13,000 rpm for 30 min at 4 °C. Samples were separatedusing SDS-PAGE before transferring to nitrocellulose membranes. Blots were blocked with 5% BSA for 2 h, and incubated with the appropriate primary antibodies over night at 4 °C (anti-PKA protein kinase catalytic subunit 1:1000; anti-PDE4A5 1:1000; anti-PDE4B1 1:1000; anti-PDE4D3 1:1000; anti-IL-1β, 1:1000; anti-IL-6, 1:1000; anti-Bcl-2, 1:1000; anti-Bax, 1:1000; anti-pCREB, 1:1000; anti-CREB, 1:1000; anti-BDNF, 1:1000 and anti-β-actin, 1:5000). Then the membranes wereincubated with the secondary antibodies (anti-mouse lgG or anti-rabbit lgG) for 60 min at room temperature. After rinsing with buffer, the immunocomplexes were visualized by chemiluminescence using the ECL kit according to the manufacturer's instructions. The film signals were digitally scanned and then for sequent analysis with software.

### Data analysis

All data were expressed as means ± standard error of the mean (SEM). All data were analyzed using one-way analysis of variance (ANOVA) except for the data from the acquisition training of the Morris water maze, which were analyzed using two-way ANOVA, followed by a post hoc Dunnett's test. Difference with *p* < 0.05 were considered statistically significant.
